# Recurrence rate of cholecystitis after initial gallbladder stenting versus secondary gallbladder stenting: A propensity score matching study

**DOI:** 10.1002/deo2.70047

**Published:** 2024-12-26

**Authors:** Ryota Nakabayashi, Hideki Kamada, Masahiro Ono, Toshiaki Kono, Naoki Fujita, Hiroki Yamana, Kiyoyuki Kobayashi, Joji Tani, Yasuhisa Ando, Hironobu Suto, Minoru Oshima, Keiichi Okano, Hideki Kobara

**Affiliations:** ^1^ Department of Gastroenterology and Neurology Institute of Medicine Kagawa University Kagawa Japan; ^2^ Division of Innovative Medicine for Hepatobiliary and Pancreatology Institute of Medicine Kagawa University Kagawa Japan; ^3^ Department of Gastroenterological Surgery Institute of Medicine Kagawa University Kagawa Japan

**Keywords:** acute, cholangiopancreatography, cholecystitis, endoscopic gallbladder drainage, endoscopic gallbladder stenting, endoscopic retrograde, gallbladder

## Abstract

**Objective:**

Limitations are sometimes encountered in the application of laparoscopic cholecystectomy to the treatment of acute cholecystitis. Endoscopic gallbladder stenting (EGBS) has emerged as an additional option. However, the long‐term stent patency remains an issue. This study was performed to compare the efficacy of primary and secondary EGBS.

**Methods:**

Sixty‐one patients who underwent preplanned EGBS because of poor surgical tolerance from January 2006 to July 2023 were retrospectively analyzed. The patients were divided into the initial EGBS group, in which EGBS was performed as the first option (*n* = 37), and the secondary EGBS group, in which EGBS was performed following other treatments (*n* = 24). The primary endpoint was the 3‐month recurrence rate, and the secondary endpoint was the technical success rate. Propensity score matching was performed to align the patients’ background factors between the two groups.

**Results:**

After propensity score matching, six patients from each group were selected for analysis. The technical success rate was significantly higher in the secondary EGBS group (73.0% [27/37] vs. 95.8% [23/24], respectively). Furthermore, the 3‐month recurrence rate was significantly higher in the initial than secondary EGBS group (66.7% [4/6] vs. 0.0% [0/6], respectively; *p* = 0.0232).

**Conclusion:**

Secondary EGBS may effectively prevent recurrent cholecystitis in patients with poor surgical tolerance.

## 1 INTRODUCTION

Emergency and early elective laparoscopic cholecystectomy plays an important role in the treatment of acute cholecystitis, as described in the Tokyo Guidelines 2018.^1^ However, the aging patient population and the prevalence of underlying diseases present challenges for surgery.^2^, ^3^ Nonsurgical treatment of acute cholecystitis includes conservative treatment with antimicrobial agents, percutaneous transhepatic gallbladder drainage/aspiration (PTGBD/A), and endoscopic gallbladder stenting (EGBS); recently, endoscopic ultrasonography‐guided gallbladder drainage (EUS‐GBD) has also shown good results.^4^, ^5^, ^6^ Although gallbladder drainage (excluding conservative treatment) shows good short‐term improvement, recurrence remains problematic when elective surgery is difficult.^7^, ^8^

For patients with acute cholecystitis, PTGBD has long been the first choice for gallbladder drainage either when surgery is difficult or as a preoperative measure. However, PTGBD can lead to complications such as puncture‐related bleeding and peritonitis.^9^ Liu et al.^10^ also reported that PTGBD before cholecystectomy increased the surgical difficulty and incidence of perioperative complications. With growing evidence supporting the safety and efficacy of EGBS and EUS‐GBD, options for gallbladder drainage in acute cholecystitis are expanding.^9^, ^10^, ^11^, ^12^, ^13^ Inoue et al.^14^ reported that the 1‐, 3‐, and 5‐year cumulative incidence of cholecystitis was significantly lower with EGBS (3.8%, 7.2%, and 7.2%, respectively) than with PTGBD (11.7%, 17.6%, and 30.2%, respectively) and that long‐term stenting with EGBS was useful in preventing recurrent cholecystitis.

Overall, EGBS is an effective treatment for acute cholecystitis that is difficult to treat surgically. However, we have been using secondary EGBS in our daily practice to further reduce the recurrence rate. This study was performed to compare the recurrence rate of cholecystitis between patients who underwent initial and secondary EGBS.

## 2 MATERIALS AND METHODS

### 2.1 Patients

**FIGURE 1:**
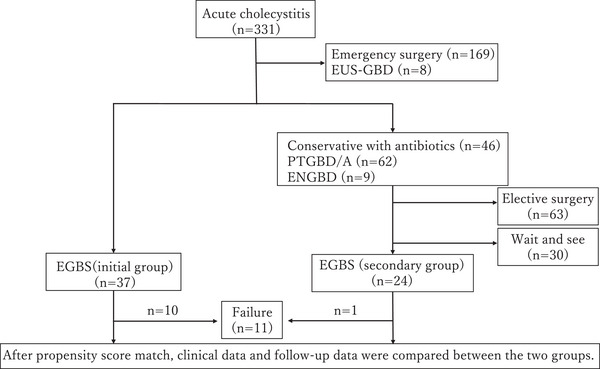
Patient flowchart.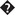 EUS‐GBD, endoscopic ultrasonography‐guided gallbladder drainage; PTGBD/A,: percutaneous transhepatic gallbladder drainage/aspiration; ENGBD, endoscopic naso‐gallbladder drainage; EGBS, endoscopic gallbladder stenting.

Information was collected from the electronic medical records of patients for whom EGBS was planned at our hospital from January 2006 to July 2023. The analysis included patients who were scheduled for EGBS as the initial treatment (I‐EGBS group) and those who were scheduled for secondary EGBS following other treatments (S‐EGBS group). Unsuccessful cases were excluded from the analysis of the cholecystitis recurrence rate. Figure 1 shows a patient flowchart. We first considered EGBS in patients who were difficult to treat with emergency surgery and who were using antithrombotic agents or had ascites effusions.

### 2.2 Study design

This was a single‐center, retrospective observational study of the above‐described patients.

### 2.3 EGBS procedure

**FIGURE 2:**
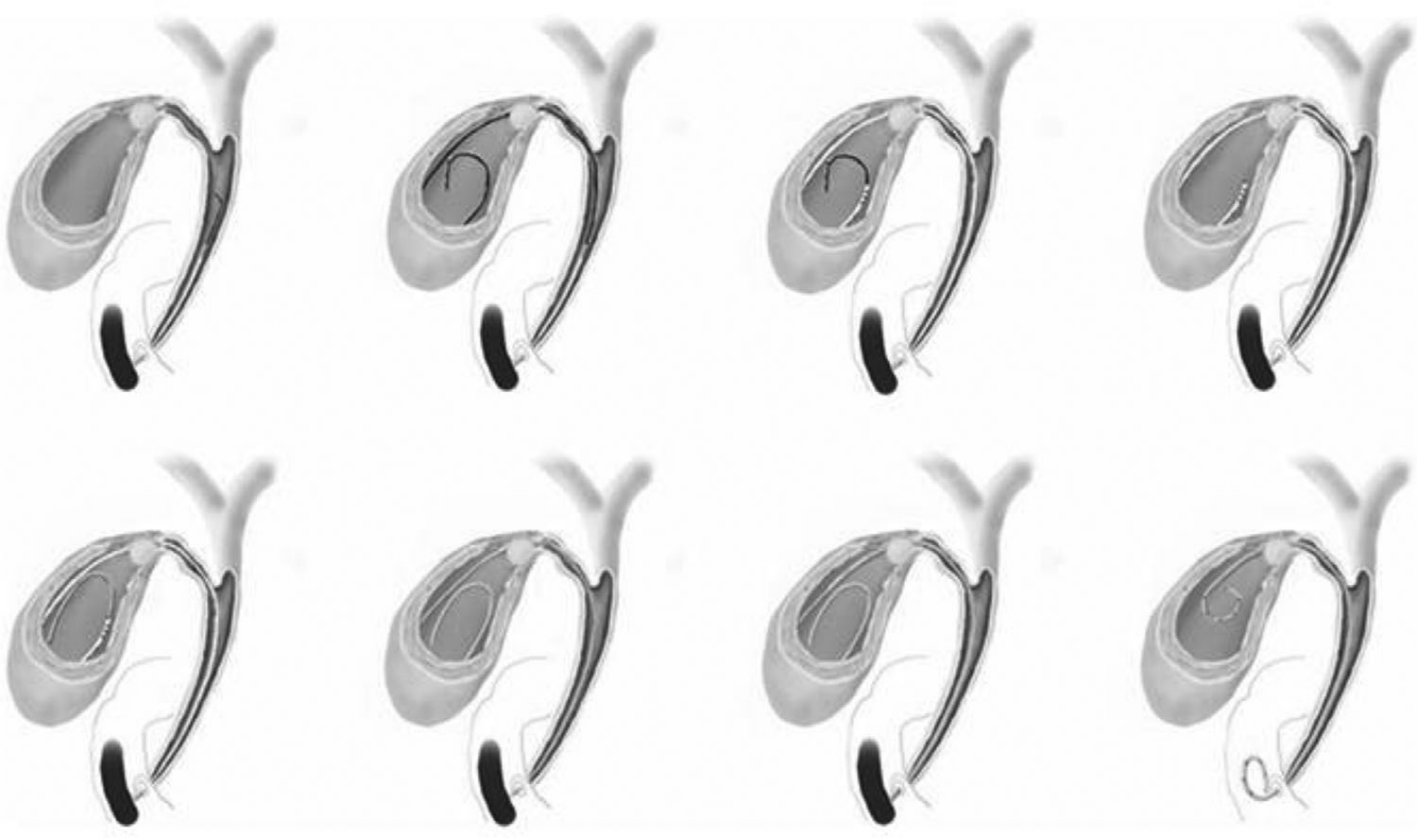
Endoscopic gallbladder stenting method. A catheter is inserted into the bile duct, and a guidewire is advanced into the gallbladder. The catheter is followed by the guidewire, and a stent is placed in the gallbladder after cholecystography is performed (cited from the *Canadian Journal of Gastroenterology and Hepatology* [15]).

**FIGURE 3:**
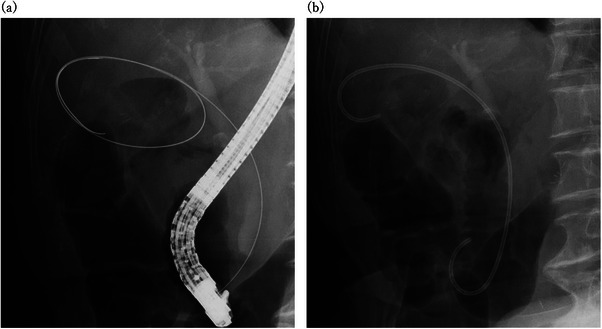
Fluoroscopic images. (a) Fluoroscopic image of gallbladder intubation. The guidewire is looped in the gallbladder. (b) Fluoroscopic image of a stent placed in the gallbladder.

An endoscope was inserted into the duodenum, and a catheter was inserted into the bile duct. After confirming the location of the choledochal duct by cholangiography, a guidewire was inserted into the gallbladder. The catheter was inserted into the gallbladder along the guidewire, and if a soft guidewire was used, it was replaced with a hard one. The procedure was terminated by placing a stent in the gallbladder along the guidewire. When endoscopic naso‐gallbladder drainage (ENGBD) was selected as the initial treatment, the ENGBD tube was pulled into the endoscope and a guidewire was inserted into the tube. The ENGBD tube was then removed, and a catheter was inserted. Thereafter, the same procedure as described above was used for stenting. Figure 2 shows the procedure, and Figure 3a,b shows the actual fluoroscopic images. All endoscopists had performed more than 100 ERCP‐related procedures.

### 2.4 Devices

#### 2.4.1 Endoscope

Duodenal videoscope (TJF‐240 [Olympus Medical Systems Corp.], TJF‐260 V [Olympus], TJF‐Q290V [Olympus], and ED‐580T [FUJIFILM Medical Co.]).

#### 2.4.2 Guidewire

Angiographic guidewire (Radifocus, 0.032‐inch [Terumo Corp.], disposable guidewire [VisiGlide 2, 0.025‐inch {Olympus}], endoscopic guidewire [VENTY, 0.025‐inch {Boston Scientific Corp.}], and guidewire for non‐vascular use [Hydra Jagwire, 0.035‐inch {Boston Scientific}]).

#### 2.4.3 Catheter

Single‐use endoscopic cannula for natural opening (MTW ERCP catheter [MTW Endoskopie Manufaktur]).

#### 2.4.4 Stent

Non‐integrated bile duct stent (Quick Place, 7‐Fr, 10–15 cm, double‐pigtail [Olympus]) and non‐integrated gallbladder stent (IYO‐stent, 5‐Fr, 32 cm [Gadelius Medical K.K.]).

### 2.5 Initial treatment protocol

**FIGURE 4:**
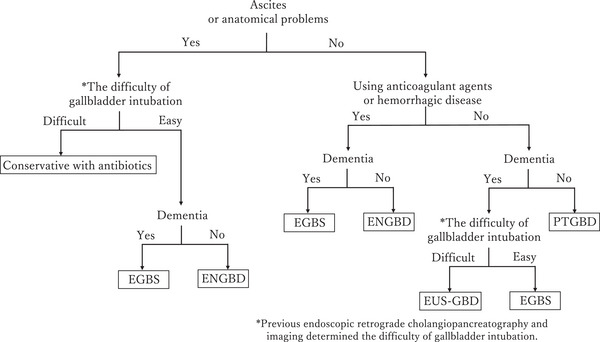
Initial treatment protocol. The treatment of patients who cannot tolerate emergency surgery was selected based on anatomical challenges that complicate percutaneous transhepatic procedures, the presence of ascites, hemorrhage risk, difficulties in intubating the choledochal tube, and the presence of dementia. The degree of difficulty of choledochal intubation was assessed using contrast‐enhanced computed tomography or prior fluoroscopic images when there was a history of endoscopic retrograde cholangiopancreatography.

The cholecystitis treatment protocol in patients who were not candidates for emergency surgery is outlined in Figure 4. Treatment options were based on factors such as ascites, anatomical challenges, the use of antithrombotic medications, susceptibility to bleeding, dementia, and difficulty with cholecystostomy. Difficulty with choledochal intubation was assessed using contrast‐enhanced computed tomography and previous fluoroscopic imaging, particularly in patients with a history of ERCP.

If a patient had ascites or anatomical barriers to percutaneous transhepatic intervention and choledochal intubation was expected to be difficult, conservative treatment with antimicrobial agents was preferred as the first option. If cholecystostomy was deemed feasible, endoscopic gallbladder drainage was chosen. Specifically, EGBS was used for patients with dementia to prevent self‐extraction due to cognitive decline, while ENGBD was selected for patients without dementia.

For patients at high risk of bleeding but without ascites or anatomical difficulties, endoscopic gallbladder drainage remained the preferred option. PTGBD was considered for patients without ascites and with anatomical suitability for percutaneous transhepatic procedures, provided they were at low risk of bleeding and did not have dementia. For patients with dementia, EGBS was selected if choledochal intubation was feasible, while EUS‐GBD was preferred if intubation was likely to be difficult. When any of these treatments failed, alternative options, including PTGBA, were selected as appropriate.

### 2.6 Outcome measures

The primary outcome was the 3‐month cholecystitis recurrence rate. The secondary outcome was the technical success rate of the procedures. Recurrence was defined as the return of symptoms, physical examination findings, and blood test abnormalities after initial improvement. Technical success was defined as successful stent implantation.

### 2.7 Statistical methods

Propensity score matching and the Kaplan–Meier (log‐rank) test were performed using EZR. Statistical analyses were performed using GraphPad Prism version 9 (GraphPad Software). Propensity score matching was performed using one‐to‐one nearest‐neighbor matching without replacement. The caliper width was set to 0.005 so that the standardized mean difference for each item was <0.1. Depending on the variable, significant difference tests were performed using the Mann–Whitney test, chi‐square test, and Fisher's exact test, depending on the item. A *p*‐value of < 0.05 was considered statistically significant.

## 3 RESULTS

### 3.1 Patients’ background factors

EGBS was planned for 61 patients (I‐EGBS group, *n* = 37; S‐EGBS group, *n* = 24). However, there were significant differences between the two groups in cholecystitis severity (Tokyo Guidelines grade) and age (Table 1). Therefore, to balance the background factors, propensity score matching was performed to obtain a standardized mean difference of <0.1 for age, sex, and cholecystitis severity. Only successful cases from both groups were included in the matching process. After matching, both groups were reduced to six patients each, and the previously significant differences in patient background factors were no longer present. In the S‐EGBS group, conservative treatment was the most common initial treatment (three patients [50.0%]), followed by PTGBD in two (33.3%) patients and ENGBD in one (16.7%) patient (Table 2).

**TABLE 1:** Patients’ clinical background factors before propensity score matching.

	Total (*n* = 61)	I‐EGBS (*n* = 37)	S‐EGBS (*n* = 24)	SMD
Age, median (range), years	77 (50–94)	77.5 (72–86)	82.5 (60–93)	0.800
Sex, male: female, *n*	37:24	22:15	15:9	0.062
Severity grade of Tokyo Guidelines 2018, *n* Grade I Grade II Grade III	24 28 9	18 10 9	6 18 0	1.224
Initial treatment before EGBS, *n* (%) Conservative with antibiotics PTGBD ENGBD			14 (58.3) 8 (33.3) 2 (8.3)	
Cause of cholecystitis, *n* (%) Gallbladder stones Cholecystic duct stones Common bile duct stones Cholecystic carcinoma Cholangiocarcinoma Plastic stent Gallbladder bleeding	40 (65.6) 8 (13.1) 9 (14.8) 1 (1.6) 1 (1.6) 1 (1.6) 1 (1.6)	20 (54.1) 8 (21.6) 6 (16.2) 1 (2.7) 0 (0.0) 1 (2.7) 1 (2.7)	20 (83.3) 0 (0.0) 3 (12.5) 0 (0.0) 1 (4.2) 0 (0.0) 0 (0.0)	1.010

Abbreviations: ENGBD, endoscopic naso‐gallbladder drainage; I‐EGBS, initial endoscopic gallbladder stenting; PTGBD, percutaneous transhepatic gallbladder drainage; S‐EGBS, secondary endoscopic gallbladder stenting; SMD, standardized mean difference.

**TABLE 2:** Patients’ clinical background factors after propensity score matching.

	Total (*n* = 12)	I‐EGBS (*n* = 6)	S‐EGBS (*n* = 6)	SMD
Age, median (range), years	76.5 (68–86)	77.5 (72–86)	77.7 (68–85)	0.027
Sex, male: female, *n*	8:4	4:2	4:2	<0.001
Severity grade of Tokyo Guidelines 2018, *n* Grade I Grade II Grade III	4 8 0	2 4 0	2 4 0	<0.001
Initial treatment before EGBS, *n* (%) Conservative with antibiotics PTGBD ENGBD			3 (50.0) 2 (33.3) 1 (16.7)	
Cause of cholecystitis, *n* (%) Gallbladder stones Cholecystic duct stones Common bile duct stones Cholecystic carcinoma Cholangiocarcinoma Plastic stent Gallbladder bleeding	12 (100.0) 0 (0.0) 0 (0.0) 0 (0.0) 0 (0.0) 0 (0.0) 0 (0.0)	6 (100.0) 0 (0.0) 0 (0.0) 0 (0.0) 0 (0.0) 0 (0.0) 0 (0.0)	6 (100.0) 0 (0.0) 0 (0.0) 0 (0.0) 0 (0.0) 0 (0.0) 0 (0.0)	<0.001

Abbreviations: ENGBD, endoscopic naso‐gallbladder drainage; I‐EGBS, initial endoscopic gallbladder stenting; PTGBD, percutaneous transhepatic gallbladder drainage; S‐EGBS, secondary endoscopic gallbladder stenting; SMD, standardized mean difference.

In the I‐EGBS group (37 patients), the most common cause of cholecystitis was gallbladder stones (20 patients), followed by obvious cholecystic duct stones (eight patients). Cholecystitis due to common bile duct stones was present in six patients, cholecystic carcinoma in one, a stray plastic stent placed for cholangiocarcinoma in one, and gallbladder bleeding of unknown origin in one. Cholangitis secondary to common bile duct stones was a complication in eight patients. In the S‐EGBS group (24 patients), the most common cause of cholecystitis was gallbladder stones (20 patients). Cholecystitis due to common bile duct stones was present in three patients, and cholangiocarcinoma in one. Cholangitis secondary to common bile duct stones was a complication in three patients (Table 1). After propensity score matching, all patients in both groups had acute cholecystitis due to gallbladder stones (Table 2).

### 3.2 Outcomes

The overall number of successful procedures was 50 of 61 (82.0%), with 27 of 37 (73.0%) in the I‐EGBS group and 23 of 24 (95.8%) in the S‐EGBS group. The success rate was significantly higher in the S‐EGBS group (*p* = 0.038). Among the unsuccessful cases, five patients were treated with PTGBD, four with conservative treatment using antimicrobial agents, and two with emergency surgery to address cholecystitis (Table 3). In the I‐EGBS group, four unsuccessful cases were due to the inability to access the cholecystic duct with a guidewire. In two cases, the guidewire was inserted into the cholecystic duct but could not bypass a stone lodged in the duct. In two other cases, the guidewire was successfully inserted into the gallbladder, but advancing the catheter was difficult. Additionally, two failures were due to perforation of the duct during the procedure. The single unsuccessful case in the S‐EGBS group occurred after conservative treatment, where guidewire access to the cholecystic duct was not possible. The median procedure time was significantly shorter in the S‐EGBS group (40 min; interquartile range [IQR]: 31.75–51.5) than in the I‐EGBS group (69 min; IQR: 52–92; *p* < 0.0001). However, after propensity score matching, the median times in the I‐EGBS and S‐EGBS groups were 52.5 min (IQR: 40–131) and 36.5 min (IQR: 26.25‐46.75), respectively, with no significant difference (*p* = 0.1797).

**TABLE 3:** Technical success rates, causes of unsuccessful cases, procedure times, alternative treatments, and adverse events

	Total (*n* = 61)	I‐EGBS (*n* = 37)	S‐EGBS (*n* = 24)	*p*
Technical success, *n* (%)	50 (82.0)	27 (73.0)	23 (95.8)	0.038*
Causes of unsuccessful cases, *n* Unable to access CD with GW Unable to bypass stone with GW Difficulty in advancing catheter Perforation of CD	5 2 2 2	4 2 2 2	1 0 0 0	0.7244
Procedure time, median (IQR), minutes	56 (39–72)	69 (52–92)	40 (31.75–51.5)	<0.0001*
Alternative treatment, *n* PTGBD Conservative with antibiotics Emergency surgery	5 4 2	4 4 2	1 0 0	0.5169
Adverse events, *n* Cholangitis Perforation of CD Pancreatitis Stent deviation CBD stones	11 3 2 1 3 2	8 3 2 0 1 2	3 0 0 1 2 0	0.502

Abbreviations: CBD, common bile duct; CD, cholecystic duct; GW, guidewire; I‐EGBS, initial endoscopic gallbladder stenting; IQR, interquartile range; PTGBD, percutaneous transhepatic gallbladder drainage; S‐EGBS, secondary endoscopic gallbladder stenting.

*p*‐Values were calculated by comparing the I‐EGBS and S‐EGBS groups according to Fisher's exact test or the chi‐square test.

* Statistically significant difference.

**FIGURE 5:**
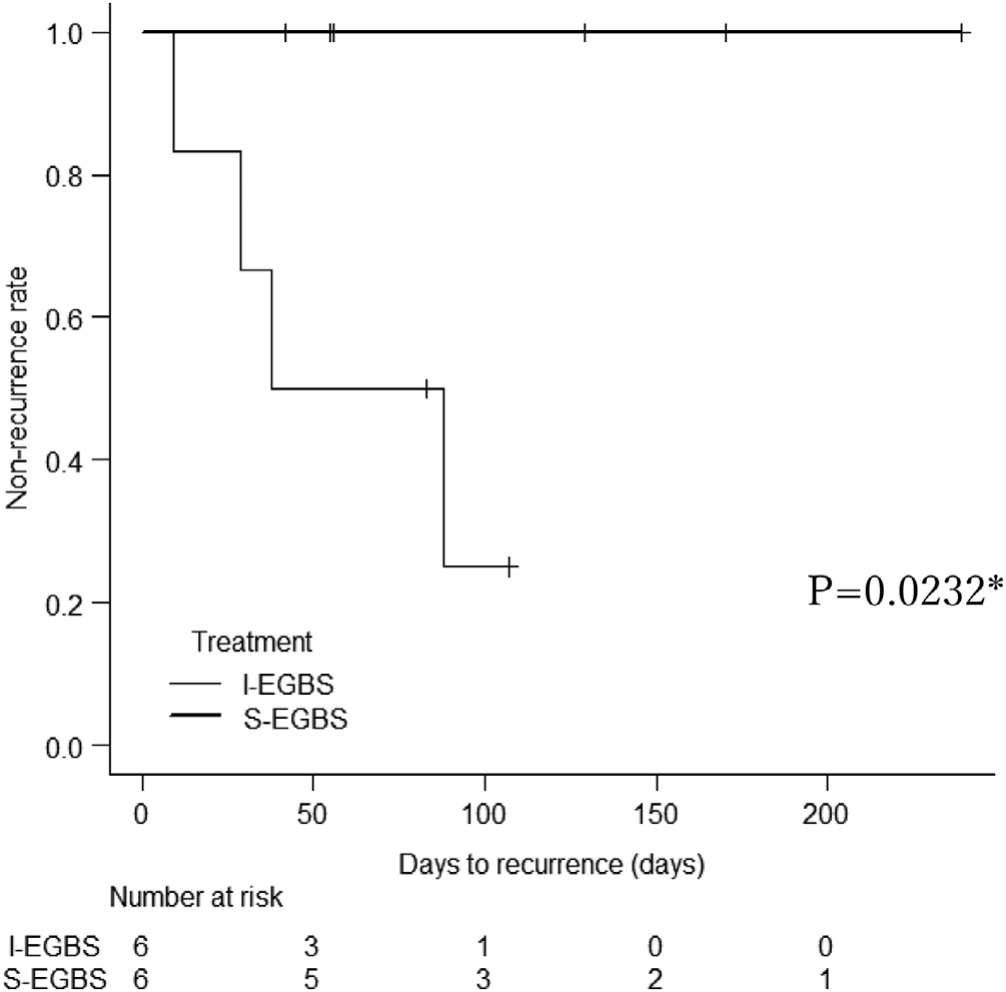
Kaplan–Meier curve for recurrence of cholecystitis after propensity score matching. The recurrence rate of cholecystitis 3 months after endoscopic gallbladder stenting (EGBS) was significantly lower in the secondary EGBS (S‐EGBS) group than in the initial EGBS (I‐EGBS) group (*p* = 0.0232).

Kaplan–Meier analysis was conducted to evaluate the time to recurrence after propensity score matching, showing that the recurrence rate at 3 months post‐EGBS was significantly lower in the S‐EGBS group (*p* = 0.0232; Figure 5). Additional Kaplan–Meier analysis for patients who underwent successful EGBS prior to propensity score matching yielded a p‐value of 0.014, further indicating that the S‐EGBS group had a significantly lower cholecystitis recurrence rate (Figure S1). The median time to recurrence was 33.5 days (range: 9–88 days), and the median duration of observation, including the date of recurrence, was 69.5 days (range: 9–239 days; 60.5 days [range: 9–107 days] in the I‐EGBS group and 92.5 days [range: 42–239 days] in the S‐EGBS group). There was no significant difference in the observation duration between the two groups (*p* = 0.1797).

Procedure‐related early complications occurred in one of 12 (8.3%) patients. There was no significant difference between the I‐EGBS group [1/6 (16.7%)] and the S‐EGBS group [0/6 (0.0%); *p* > 0.9999; Table 4). Contingencies were also examined in both groups before propensity score matching, including unsuccessful cases: eight in the I‐EGBS group and three in the S‐EGBS group, with no significant difference in incidence (21.6% vs. 12.5%, respectively). Table 3 provides a breakdown of these contingencies. Early accidental complications included cholangitis, while late complications included stent deviation and the formation of common bile duct stones.

**TABLE 4:** Observational period, cholecystitis recurrence rate, time to recurrence, and adverse events

	Total (*n* = 12)	I‐EGBS (*n* = 6)	S‐EGBS (*n* = 6)	*p*
Number of recurrent cholecystitis cases at 3 months, *n* (%)	4 (33.3)	4 (66.7)	0 (0.0)	0.0232*
Duration until recurrence, median (range), days	33.5 (9–88)	33.5 (9–88)	–	–
Observational period, median (range), days	69.5 (9–239)	60.5 (9–107)	92.5 (42–239)	0.1797
Adverse events, *n* Cholangitis	1 1	1 1	0 0	>0.9999

Abbreviations: I‐EGBS, initial endoscopic gallbladder stenting; S‐EGBS, secondary endoscopic gallbladder stenting.

*p*‐Values were calculated by comparing the I‐EGBS and S‐EGBS groups according to the log‐rank test, Mann–Whitney test, or Fisher's exact test.

* Statistically significant difference.

Stent diameters varied between 7‐ and 5‐Fr depending on the time of year: 22 patients in both the I‐EGBS and S‐EGBS groups received 7‐Fr stents, while 5 patients in the I‐EGBS group and 1 in the S‐EGBS group received 5‐Fr stents. Cholecystitis recurred in all patients with 7‐Fr stenting, with a 3‐month recurrence rate of 13.6% for the 7‐Fr stent compared with 0.0% for the 5‐Fr stent. However, no significant difference was found between the two groups (*p* > 0.9999).

Endoscopic sphincterotomy (EST) was performed in 15 patients in the I‐EGBS group and 4 in the S‐EGBS group, with no EST‐related complications (such as bleeding) observed. The cholecystitis recurrence rate was significantly higher in patients who did than did not undergo EST (five of 19 (26.3%) vs. one of 31 [3.2%] patients, respectively; *p* = 0.0244).

Among the eight cases of successful I‐EGBS, there were no perioperative complications, and all surgeries were completed successfully. In one case, although laparoscopic surgery was planned, the strong adhesion between the omentum and gallbladder necessitated a laparotomy. For the successful S‐EGBS cases, elective surgery was performed in two instances, with no notable perioperative issues (Figure S2).

## 4 DISCUSSION

Our previous study demonstrated a low recurrence rate of acute cholecystitis when EGBS was performed following initial therapy.^15^ In this study, there were no cases of recurrence within the 3‐month observation period. Furthermore, the 3‐month cholecystitis recurrence rate was significantly lower in the S‐EGBS than in the I‐EGBS group. The main reason for this difference may be the effective clearance of bile sludge and bacteria from the gallbladder following the initial treatment. Additionally, because a stent was placed afterward, it may have remained patent for a longer duration. The median time to cholecystitis recurrence in the I‐EGBS group was approximately 1 month, suggesting that even if elective laparoscopic cholecystectomy is selected after EGBS, the patient may relapse before the scheduled surgery date. Secondary EGBS may therefore be valuable, particularly as more patients with acute cholecystitis are advised to avoid emergency surgery. Although S‐EGBS has the potential to reduce recurrence after treatment of acute cholecystitis, this treatment approach may inevitably result in a longer hospital stay. Additionally, compared with I‐EGBS, the number of percutaneous and endoscopic procedures increases, leading to greater treatment invasiveness and higher medical costs. However, if cholecystitis recurs repeatedly, the invasiveness and costs associated with treatment will also rise after I‐EGBS. Therefore, prospective studies are needed to evaluate these factors.

The success rate of EGBS reportedly ranges from 72.8% to 100%.^15^, ^16^, ^17^, ^18^, ^19^, ^20^, ^21^, ^22^, ^23^, ^24^ In this study, both groups demonstrated similarly high success rates, consistent with previous findings. Several factors may have contributed to the high procedural success in the S‐EGBS group. In the I‐EGBS group, most unsuccessful cases were due to difficulty in accessing the cholecystic duct with a guidewire. In these instances, the cholecystic duct could not be visualized through contrast from the bile duct and had to be accessed blindly. This issue may be attributed to increased gallbladder pressure, which was less likely in the S‐EGBS group because the gallbladder pressure had decreased following the initial treatment. Additionally, the overall procedure time was significantly shorter in the S‐EGBS group. This may be attributed to the reduced intracholecystic pressure, which facilitated contrast‐enhanced visualization. Another contributing factor may be that some patients in the I‐EGBS group underwent common bile duct stone removal in conjunction with EGBS.

Pancreatitis, cholangitis, and perforation of the cystic duct were observed in early cases. Only one case of cholangitis required additional EST, while all other cases improved with conservative treatment alone. In later cases, stent deviation and common bile duct stones were noted; however, stent deviation did not pose a problem for any of the patients. The observation end date was recorded as the date when the deviation was detected. In cases where new common bile duct stones were found, removal and bile duct drainage were necessary; however, these events were not associated with cholecystitis recurrence and did not impact the course of cholecystitis. Although no significant difference was observed between the two groups, the incidental findings related to stent placement warrant attention.

The stents used in this study had two diameters: 7‐ and 5 Fr. As a subanalysis, we examined the difference in cholecystitis recurrence rates based on stent diameter and found no significant difference between the two groups. However, the number of cases was limited, and the results may change if the number of 5‐Fr stent cases increases.

There are two potential reasons for the increased cholecystitis recurrence rate following EST. First, the loss of papillary function after EST may facilitate retrograde infection, although this may not be a major concern because stents were placed regardless of the EST status. Second, EST was performed more frequently in the I‐EGBS group. EST is typically conducted for stone treatment in patients with common bile duct stones, and EGBS is performed immediately after stone treatment. Therefore, the recurrence rate in the I‐EGBS group may have influenced the recurrence rate among patients who underwent EST.

Although we believe we were able to show good results for secondary EGBS, the present study had three main limitations. First, the sample size was small. Second, it was a single‐center retrospective study. Because of these two limitations, propensity score matching was required to prevent bias due to differences in the patients’ background factors. Although the background factors of the two groups were well matched, the proportion of Grade I and III cases was lower than in the overall population. However, the overall 3‐month recurrence rate was still significantly higher in the I‐EGBS group. Therefore, we do not believe that these factors substantially impacted the evaluation of the primary endpoint. Nonetheless, factors such as length of hospitalization, which were not examined in this study, might have an effect, and careful evaluation of these factors may be necessary.

The Tokyo Guidelines 2018 emphasize the necessity of intervention in cases of Grade ≥II acute cholecystitis.^1^ In this study, the low recurrence rate of cholecystitis in the S‐EGBS group suggests that secondary EGBS may be a viable option for inoperable cases. However, long‐term stenting carries risks, including the recurrence of common bile duct stones and the formation of stent‐stone complexes. Further investigation is ongoing to compare cholecystitis recurrence rates between long‐term stenting and other treatment modalities, as well as to evaluate the necessity of periodic stent replacement.

In conclusion, secondary EGBS may be an effective option for preventing recurrent cholecystitis in patients with poor surgical tolerance.

## CONFLICT OF INTEREST STATEMENT

Hideki Kobara, a co‐author, is the associate editor of *DEN Open*.

## ETHICS STATEMENT

Approval of the Research Protocol: This study was ethically reviewed and approved by the Ethical Review Committee of Kagawa University (2024‐053).

## PATIENT CONSENT STATEMENT

As an alternative to traditional informed consent, the patients were provided information and allowed to refuse the use of their medical record information.

## CLINICAL TRIAL REGISTRATION

2024–053.

## Supporting information

Supplementary Figure 1
Supplementary Figure 2
