# Supplementary figures and images for: Recurrence rate of cholecystitis after initial gallbladder stenting versus secondary gallbladder stenting: A propensity score matching study
Source: DEN Open. 2024 Dec 26;5(1):e70047. doi: 10.1002/deo2.70047 (PMC11670053; doi:10.1002/deo2.70047)

Supplementary Figure 1.

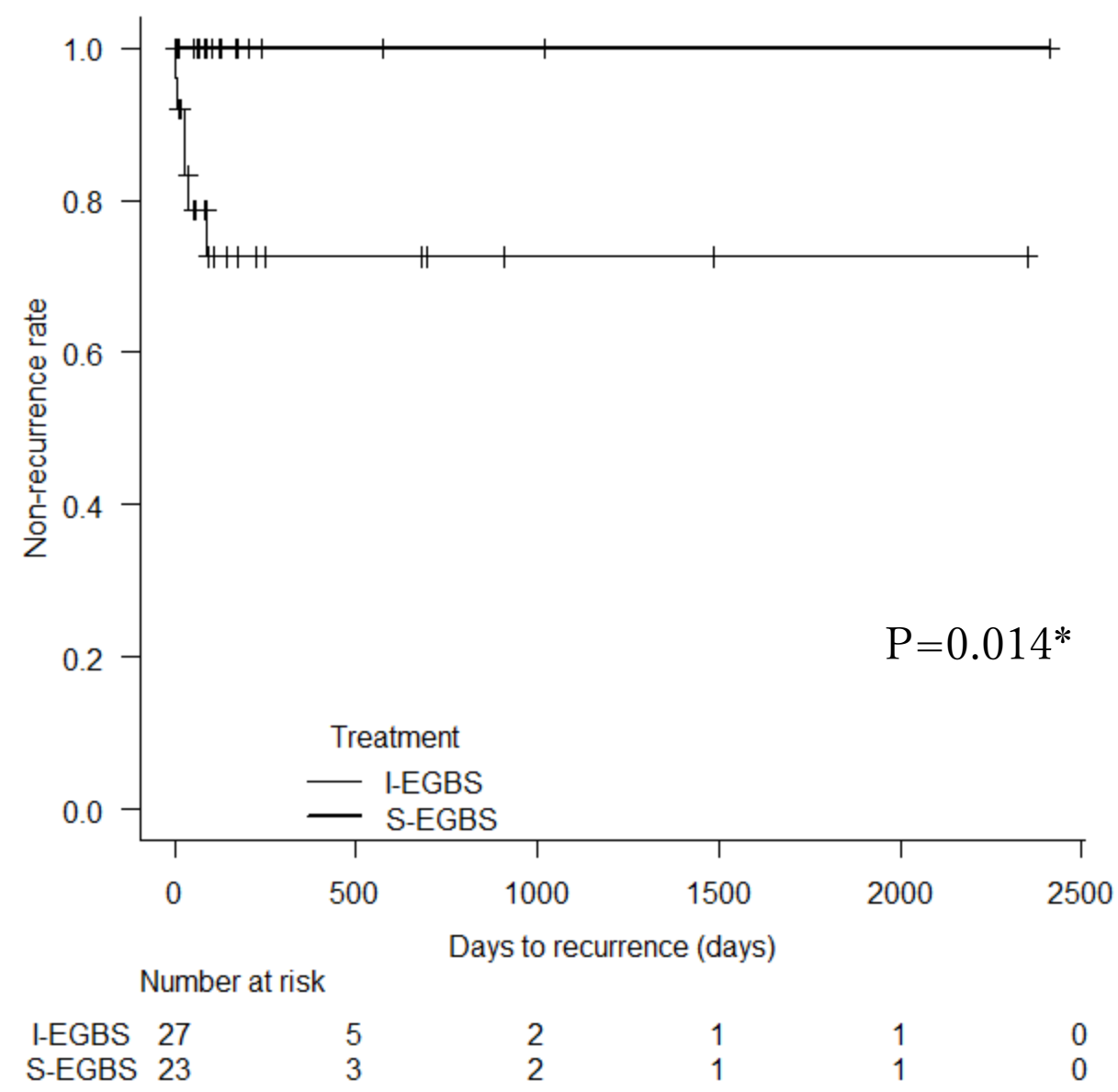

Supplementary Figure 2.

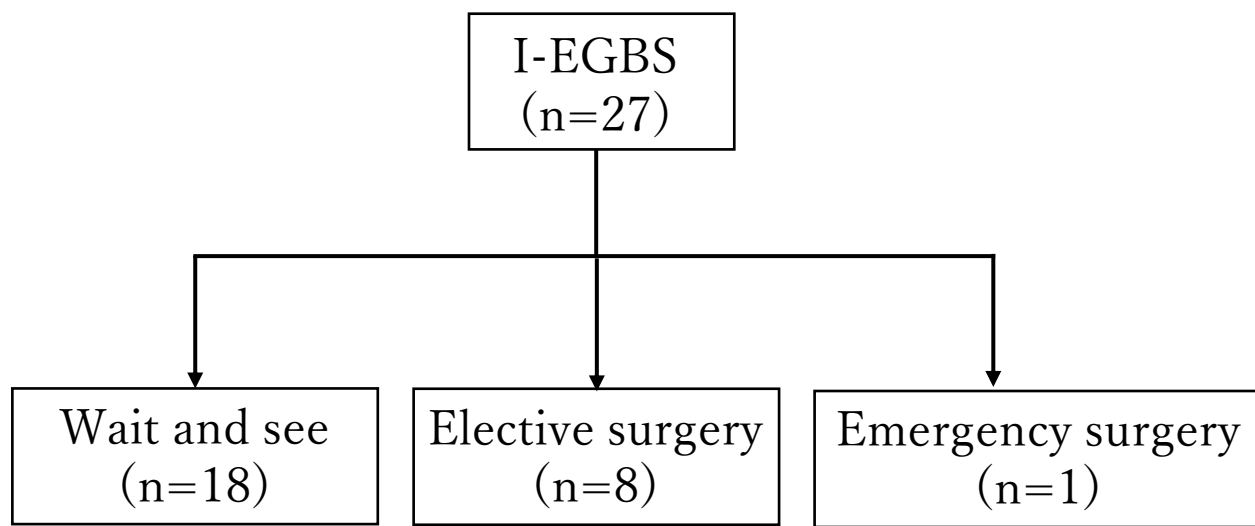

\*In the event of a recurrence

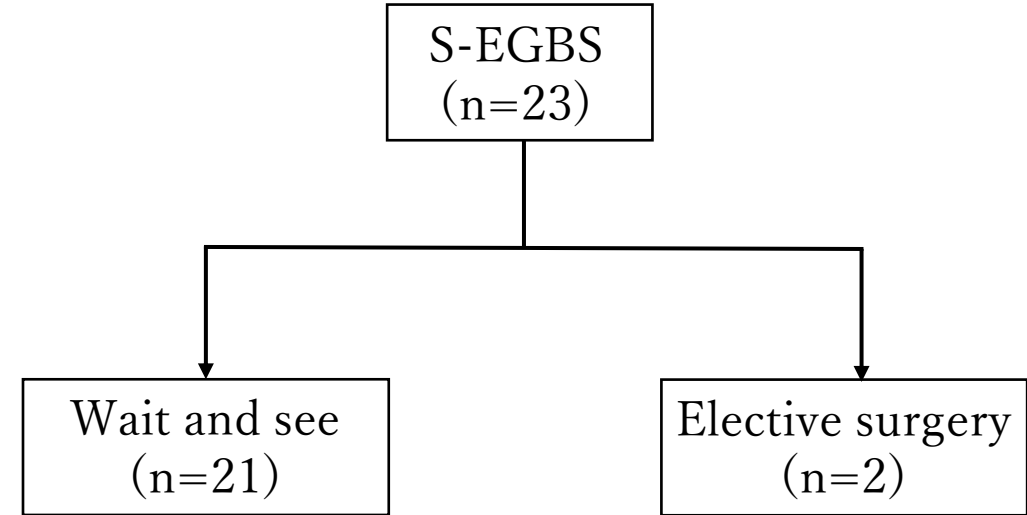

Supplement: Supplementary file 1 — Supplementary Figure 1 Supplementary Figure 2 [file DEO2-5-e70047-s001.pdf]
